# Soluble CD146 as a Potential Target for Preventing Triple Negative Breast Cancer MDA-MB-231 Cell Growth and Dissemination

**DOI:** 10.3390/ijms23020974

**Published:** 2022-01-17

**Authors:** Akshita Sharma, Ahmad Joshkon, Aymen Ladjimi, Waël Traboulsi, Richard Bachelier, Stéphane Robert, Alexandrine Foucault-Bertaud, Aurélie S. Leroyer, Nathalie Bardin, Indumathi Somasundaram, Marcel Blot-Chabaud

**Affiliations:** 1 Department of Stem Cell and Regenerative Medicine, D.Y. Patil Universit, Kolhapur 416003, India; sharmaakshita237@gmail.com (A.S.); drindumathisomasundaram@gmail.com (I.S.); 2 Faculty of Pharmacy, Aix-Marseille University, INSERM 1263, INRAE 1260, C2VN, 13005 Marseille, France; ahmad.joshkon@univ-amu.fr (A.J.); aymen.ladjimi@univ-amu.fr (A.L.); wael.traboulsi@univ-amu.fr (W.T.); richard.bachelier@univ-amu.fr (R.B.); stephane.robert@univ-amu.fr (S.R.); alexandrine.bertaud@univ-amu.fr (A.F.-B.); aurelie.leroyer@univ-amu.fr (A.S.L.); nathalie.bardin@univ-amu.fr (N.B.)

**Keywords:** CD146, triple negative breast cancer, treatment

## Abstract

**Background:** Triple Negative Breast Cancers (TNBC) are the most aggressive breast cancers and lead to poor prognoses. This is due to a high resistance to therapies, mainly because of the presence of Cancer Stem Cells (CSCs). Plasticity, a feature of CSCs, is acquired through the Epithelial to Mesenchymal Transition (EMT), a process that has been recently shown to be regulated by a key molecule, CD146. Of interest, CD146 is over-expressed in TNBC. **Methods:** The MDA-MB-231 TNBC cell line was used as a model to study the role of CD146 and its secreted soluble form (sCD146) in the development and dissemination of TNBC using in vitro and in vivo studies. **Results:** High expression of CD146 in a majority of MDA-MB-231 cells leads to an increased secretion of sCD146 that up-regulates the expression of EMT and CSC markers on the cells. These effects can be blocked with a specific anti-sCD146 antibody, M2J-1 mAb. M2J-1 mAb was able to reduce tumour development and dissemination in a model of cells xenografted in nude mice and an experimental model of metastasis, respectively, in part through its effects on CSC. **Conclusion:** We propose that M2J-1 mAb could be used as an additional therapeutic approach to fight TNBC.

## 1. Introduction

CD146, also known as MUC18 or MCAM, is a 113KDa transmembrane glycoprotein that was first described by Johnson et al. as a melanoma progression antigen [1]. Later, it was found to be present in the vascular system, whatever the caliber of the vessel, in endothelial cells, smooth muscle cells and pericytes [2]. In normal cells, CD146 is also expressed by placental trophoblasts and a subset of activated T-cell [3,4].

Thereafter, CD146 has been shown to be expressed not only in melanoma but also in various cancers, such as pancreatic [5], breast [6], prostate [7], ovarian [8], hepato-carcinoma [9] and kidney [10] cancers. Its expression is generally associated with a poor prognosis for the patient. In breast cancer, CD146 induces the epithelial-to-mesenchymal transition (EMT), a crucial process in cancer metastasis [11], gaining stem cell-like properties [12] and generating cells known as Cancer Stem Cells (CSC). 

Despite CD146 having been stated to be a tumor suppressor in breast cancer in certain studies, it is noteworthy that up-modulation of CD146 is frequently related to various high-grade tumors, ER-PR negative tumors and also triple negative breast cancers (TNBC) [13]. Subsequently, its down-regulation was frequently shown to induce a less aggressive phenotype tumor [14]. 

Of interest, CD146 appears to be highly expressed in TNBC [15] and could thus constitute a novel target in this type of breast cancer for which efficient therapeutic approaches are currently lacking. However, CD146 is also vital for vascular development and functions [16]. It thus appears difficult to target tumor CD146 without affecting vascular functions. Of interest, we recently showed that CD146 expressed on cancer cells can generate a soluble form (sCD146) that is secreted and displays growth, metastatic and angiogenic properties in different cancer models [17].

Thus, in view of the high expression of CD146 in TNBC, coupled with the demonstrated role of sCD146 in cancer development, angiogenesis and dissemination, we hypothesized that sCD146 could display major effects in TNBC. We thus analyzed its role in the MDA-MB-231 cell line, which is frequently used as a model of TNBC, and proposed a new therapeutic approach targeting sCD146 to fight this cancer of poor prognosis.

## 2. Results

### 2.1. Experimental Results

#### 2.1.1. Triple Negative Breast Cancer Cells MDA-MB-231 Express Cancer Stem Cells Markers and Are Essentially Composed of Cells with High CD146 Expression

**Figure 1:**
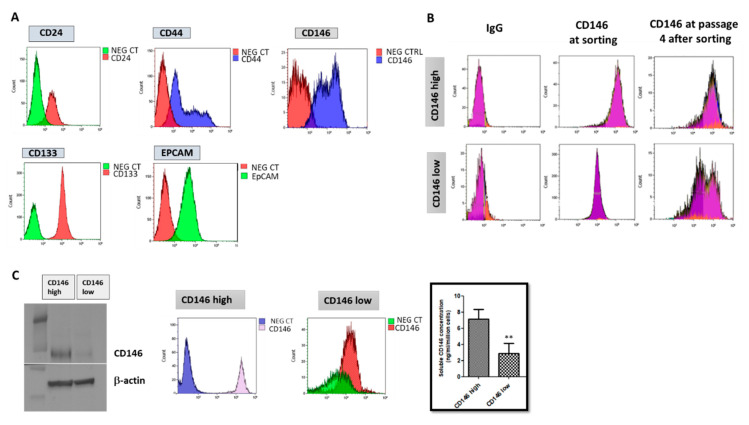
**Characterization of MDA-MB-231 cells.** (**A**) MDA-MB-231 cells were characterized for the stem cells markers CD24, CD44, CD133, EPcam and CD146 by flow cytometry. Specific antibody and irrelevant negative controls (NEG CT) were used. Representative experiments are shown. (**B**) CD146 expression was estimated by flow cytometry in CD146-high and CD146-low MDA-MB-231 cells at sorting and after culturing during four passages. A representative experiment is shown. (**C**) Cells were sorted as a function of the high or low expression of CD146, and both CD146 expression and sCD146 secretion were examined by western-blot/FACS (representative experiment) and ELISA (*n* = 6), respectively. **: *p* < 0.01, CD146 low vs. CD146 high.

The MDA-MB-231 cells were characterized for Cancer Stem Cell (CSC) markers and the CD146 surface marker population. The CSC markers studied were CD24, CD44, CD133 and EPcam. All of them were expressed by the cells with a high expression of CD44, CD133 and EPcam, and a low expression of CD24, a phenotype characteristic of breast CSC (Figure 1A). The cell surface marker CD146 was also studied. Two populations were observed with low and high expressions of CD146 in this cell line (Figure 1A). We thus sorted CD146-high and CD146-low populations for further characterization. Of interest, we found that the majority of cells were CD146-high and that, upon culturing, CD146-low cells differentiated into CD146-low and CD146-high cells, while the CD146-high cell population remained CD146-high (Figure 1B).

Thus, MDA-MB-231 cells were found to express CSC markers and to be essentially composed of CD146-high cells.

#### 2.1.2. CD146-High MDA-MB-231 Cells Secrete a High Amount of Soluble CD146 and Display Invasive Properties

To further characterize CD146-low and CD146-high cells, we performed FACS sorting of the two populations and analyzed their secretion of soluble CD146 (sCD146) by ELISA. The results (Figure 1C) shows that both populations differently expressed CD146 and that the CD146-high population secreted a higher amount of sCD146.

**Figure 2:**
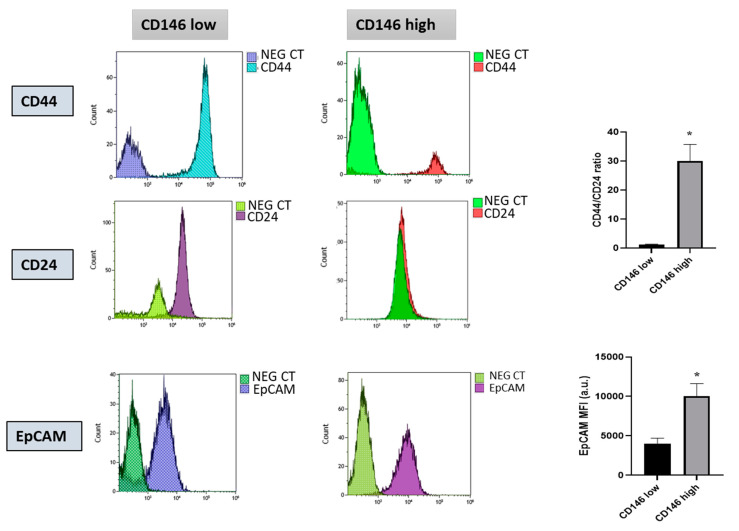
**Characterization of CD146-low and CD146-high MDA-MB-231 cells for the expression of CSC markers.** CD146-low and CD146-high MDA-MB-231 cells were characterized for the stem cells markers CD44, CD24 and EPcam by flow cytometry. Specific antibody and irrelevant negative control (NEG CT) were used. Representative experiments are shown. The average ratio of CD44/CD24 was calculated from the percentage of CD44 and CD24 positive subpopulations in the flow cytometry analysis from four different experiments. The average Mean Fluorescent Intensities (MFI) of EpCAM were determined in the two experimental conditions in four experiments. *: *p* < 0.05, CD146 high vs. CD146 low.

We also compared the CSC markers CD44, CD24 and EPcam expressed by CD146-low and CD146-high cells. We calculated the ratio of the expression level of CD44 and CD24 (CD44/CD24) from the percentage of CD44 and CD24 subpopulations in the flow cytometry analysis and showed that this ratio was significantly higher in the CD146-high population than in the CD146-low one. Likewise, EPcam expression was also significantly higher in the CD146-high population (Figure 2).

**Figure 3:**
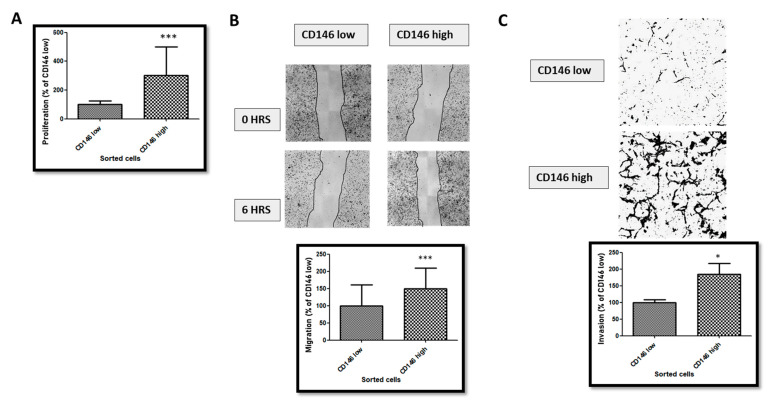
**Proliferation, migration and invasion of CD146-low versus CD146-high cells.** (**A**) Comparison of proliferation rates in low- and high- CD146 MDA-MB-231 cells. (**B**) Comparison of migration rates in low- and high- CD146 MDA-MB-231 cells. Images show representative pictures of the migration assay, and the average of five experiments is given. (**C**) Comparison of invasion rates in low- and high- CD146 MDA-MB-231 cells. Images show representative pictures of the invasion assay, and the average of five experiments is given. *, ***: *p* < 0.05, *p* < 0.001, CD146 high vs. CD146 low.

We then compared the proliferative, migratory and invasive properties of CD146-low and -high MDA-MB-231 cells. The results (Figure 3) show that CD146-high cells were more proliferative, migrative and invasive compared with the CD146-low cells.

#### 2.1.3. Soluble CD146 Stimulates MDA-MB 231 Cells Migration and Enhances Expression of Stem Cells and Epithelia-Mesenchymal Markers

**Figure 4:**
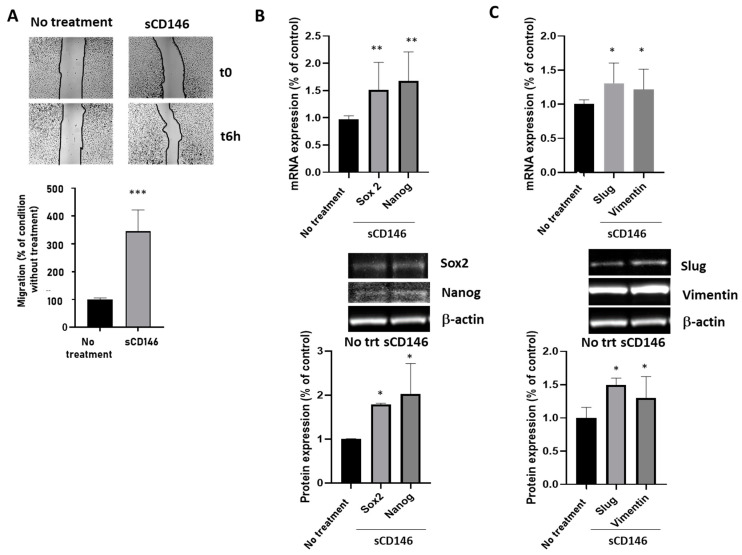
**Effect of sCD146 in CD146 high MDA-MB-231 cells.** (**A**) Effect of sCD146 100 ng/mL for 6 h on migration rates in high-CD146 MDA-MB-231 cells compared to the absence of treatment. Images show representative pictures of the migration assay, and the average of three experiments is given. (**B**) Effect of sCD146 100 ng/mL for 24 h on the expression of the CSC markers Sox2 and Nanog at the mRNA (qPCR) and protein (Western Blot) levels compared to the absence of treatment. Representative experiments and the average of four to five experiments are given. (**C**) Effect of sCD146 100 ng/mL for 24 h on the expression of the EMT markers Slug and Vimentin at the mRNA (qPCR) and protein (Western Blot) levels compared to the absence of treatment. Representative experiments and the average of four to five experiments are given. *, **, ***: *p* < 0.05, *p* < 0.01, *p* < 0.001 experimental vs. no treatment (No trt).

We analyzed the migratory properties of CD146-high MDA-MB-231 cells stimulated by sCD146. The results (Figure 4A) show that 100 ng/mL sCD146 for 24 h stimulated the migration of CD146-high cells. We also analyzed the effect of sCD146 on the expression of the CSC markers Sox2 and Nanog at the mRNA and protein levels (Figure 4B). The results show that sCD146 increased both factors. Finally, we analyzed the effect of sCD146 on the expression of Epithelial–Mesenchymal Transition (EMT) markers Slug and Vimentin at the mRNA and protein levels (Figure 4C). The results show an increase in both factors after sCD146 treatment.

#### 2.1.4. An Anti-sCD146 Monoclonal Antibody Blocks the Effects of sCD146 on MDA-MB 231 In Vitro

**Figure 5:**
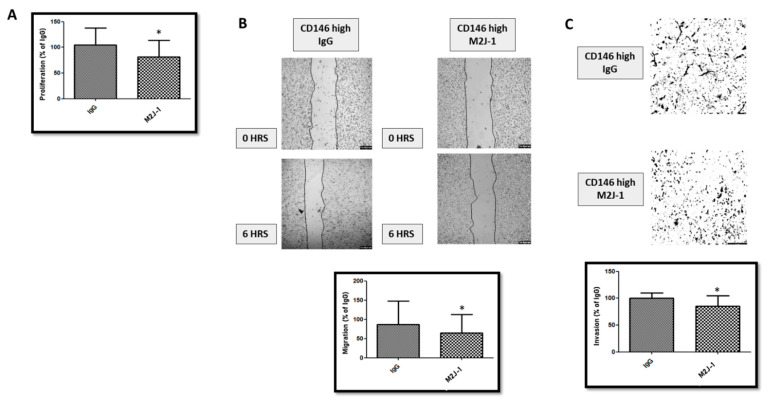
**Effect of M2J-1 antibody on the proliferation, migration and invasion in CD146 high MDA-MB-231.** (**A**) Effect of M2J-1 antibody on proliferation as compared to control IgG. (**B**) Effect of M2J-1 antibody on migration as compared to control IgG. Images show representative pictures of the migration assay, and the average of five experiments is given. (**C**) Effect of M2J-1 on invasion as compared to control IgG. Images show representative pictures of the invasion assay, and the average of five experiments is given. *: *p* < 0.05, M2J-1 vs. IgG.

**Figure 6:**
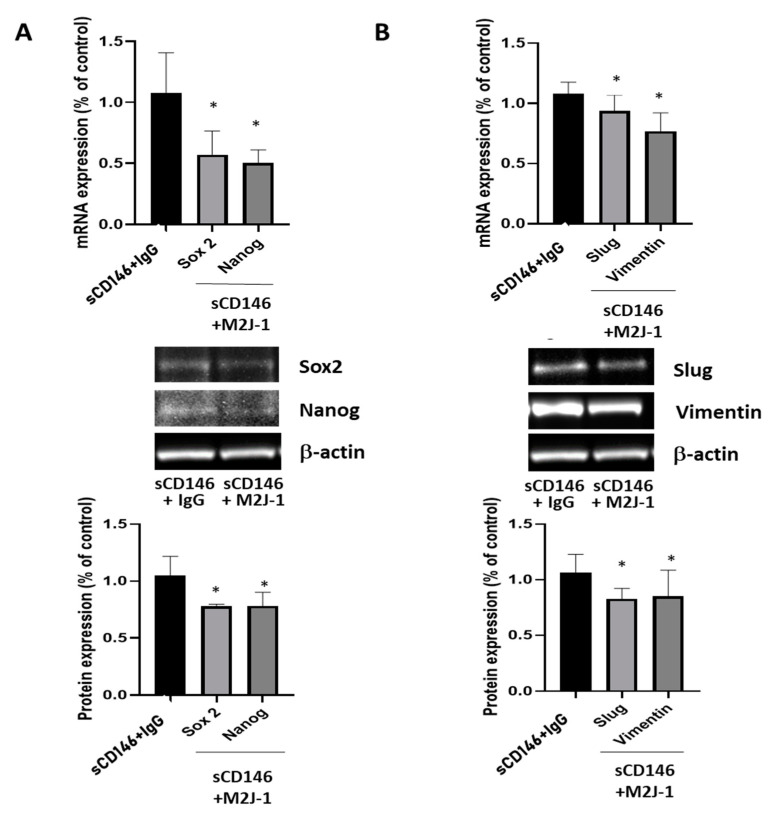
**Effect of anti-sCD146 antibody M2J-1 in CD146-high MDA-MB-231 cells**. (**A**) Effect of anti-sCD146 antibody M2J-1 50 μg/mL for 24 h as compared to control IgG on 100 ng/mL sCD146-induced increase in CSC markers Sox2 and Nanog at the mRNA (qPCR) and protein (Western Blot) levels. Representative experiments and the average of four to five experiments are given. (**B**) Effect of anti-sCD146 antibody M2J-1 50 μg/mL for 24 h as compared to control IgG on 100 ng/mL sCD146-induced increase in EMT markers Slug and Vimentin at the mRNA (qPCR) and protein (Western Blot) levels. Representative experiments and the average of four to five experiments are given. *: *p* < 0.05, sCD146 + M2J-1 vs. sCD146 + IgG.

Figure 5 shows that treatment of CD146-high MDA-MB-231 cells with 50 μg/mL of the anti-sCD146 antibody M2J-1 reduced the proliferation (A), migration (B) and invasion (C) of CD146 high MDA-MB-231 cells. In addition, Figure 6 shows that treatment of CD146-high MDA-MB-231 cells with 50 μg/mL of the anti-sCD146 antibody M2J-1 reduced the 100 ng/mL sCD146-induced an increase in expression of the CSC markers Sox2 and Nanog (A) and expression of the EMT markers Slug and Vimentin (B) compared to IgG.

#### 2.1.5. Anti-sCD146 mAb M2J-1 Prevents Tumor Growth and Metastatic Dissemination of MDA-MB-231 Cells in Two In Vivo Experimental Models

**Figure 7:**
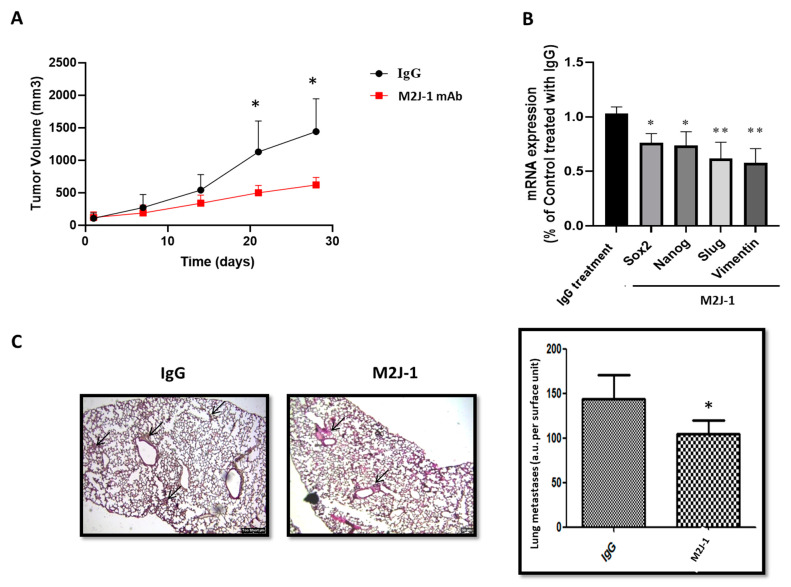
**Effect of M2J-1 mAb in two in vivo experimental models.** (**A**) Effect of anti-sCD146 antibody M2J-1 500 μg/kg compared to IgG, on tumor volume in an in vivo model of CD146-high MDA-MB-231 cells subcutaneously injected in nude mice. Four mice were used in each group. (**B**) Expression of CSC (Sox 2 and Nanog) and EMT (Slug and Vimentin) markers at the mRNA level (qPCR) in tumors from IgG and M2J-1 treated mice. (**C**) Histological analysis of lung metastases (arrows) in an experimental model of intracardiac injection of CD146-high MDA-MB-231 cells and treatment with M2J-1 mAb 500 μg/kg as compared to IgG. Six mice were used in each group. Representative experiments and the average of four to five sections for each animal are given. *, **: *p* < 0.05, *p* < 0.01, M2J-1 vs. IgG.

In order to confirm these data in vivo, two series of experiments were performed in nude mice. In a first series, the effect of 500 μg/kg M2J-1 mAb was tested in a model of nude mice subcutaneously injected with CD146-high MDA-MB-231 cells, and tumor growth was estimated in comparison with animals receiving control IgG. No side effect of the antibody injection were observed. The results (Figure 7A) show that M2J-1 mAb administration led to a decrease in tumor size. After sacrificing the animals, we analyzed the tumors for mRNA expression of CSC and EMT markers. Of interest, the results (Figure 7B) show that treatment with M2J-1 mAb led to a significant decrease in mRNA expression of Sox2 and Nanog CSC markers as well as Slug and Vimentin EMT markers compared to animals treated with IgG.

In another series of experiments, intracardiac injection of CD146-high MDA-MB-231 cells was performed in nude mice concomitantly with 500 μg/kg M2J-1 mAb or IgG a control. No side effect of the antibody injection was observed. After scarifying the animals, clustered and disfigured patches corresponding to metastases were analyzed in the lungs of animals and quantified. The results (Figure 7C) show that the total number of lung metastases was reduced in animals receiving M2J-1 mAb.

## 3. Discussion

Among breast cancers, Triple Negative Breast Cancer (TNBC) is one of the most aggressive [13] with few therapeutic options. In this study, we emphasize the role of CD146 and, in particular, its soluble form in TNBC development. Interestingly, the administration of M2J-1 mAb, which specifically targets the soluble form of CD146, led to a decrease in tumor size and metastatic dissemination and could represent a new therapeutic approach.

CD146 is considered as a major factor involved in tumor growth and dissemination. In-vivo studies have shown that its over expression leads to an increased metastatic ability of cancer cells [18]. Thus, it has been shown to increase the metastasis of lymphoma cells in a chicken model [19] and of mouse mammary carcinoma cells [20]. Wu et al. also showed that the over-expression of CD146 in prostate cancer [21] led to an increase in metastasis in-vivo [7]. CD146 has been reported to be responsible for advanced tumor stages and constitutes a poor prognosis factor for tumor relapse in ovarian cancer [22]. 

Likewise, CD146 expression is linked to a poor survival rate in pulmonary adenocarcinoma [23]. In non-small lung cancer (NSCLC) patients, CD146 expression was found to be gender specific, and its over expression leads to a poor survival rate [24]. In NSCLC, females had higher CD146 expression than males, proving it to be the poor prognostic factor for lung adenocarcinoma [25]. Finally, Peng Zeng et al. conducted a meta-analysis and showed a correlation between higher CD146 expression and poor survival rate of patients, thus, making it a valuable prognostic marker for many solid tumors [26].

In contrast to many other cancers, studies related to the correlation between CD146 and breast cancer are few and highly controversial. While some studies have shown that CD146 acts as a tumor suppressor in breast cancer because of its high expression on benign proliferative lesions and low expression on breast carcinoma [27], other studies have demonstrated a positive association between CD146 expression and breast cancer and a major role of the molecule in cell motility and invasion [14]. 

CD146 is highly expressed in TNBC [15] and is reported as a main activator of the Epithelial to Mesenchymal Transition (EMT), which constitutes an important process responsible for high metastatic features. Indeed, when over-expressed in epithelial breast cancer cells, CD146 down-regulates the epithelial markers while it up-regulates the mesenchymal markers, which significantly results in an increased cell migration and invasion ability along with an increase in Cancer Stem Cell (CSC) properties [13,14]. In addition, it has been proposed that CD146 may serve as a novel therapeutic target to overcome chemoresistance [28]. 

CD146 expression has been shown to be correlated with sCD146 secretion in many tumor cells [29], and sCD146 constitutes a major actor of tumor growth and dissemination [17]. In this study, we evidenced the effect of sCD146 on the expression of CSC markers, a phenomenon that has been frequently associated to their resistance to various treatment therapies like radiation, chemotherapy and hormonal therapy [30]. The breast cancer cell line MDA-MB-231 was used as a model in this study as it was found to have high CD146 expression. 

The cells were found to have high CD44, low CD24, high EPcam and high CD133 expression, a characteristic of breast CSC. In addition, we identified two populations of CSC with different CD146 expression, namely CD146-high and CD146-low populations as already observed by Mostert et al. [31]. We were able to sort these two populations of cells for either studying their phenotype or re-culturing them to analyze whether they were able to maintain their phenotypical characteristics. 

We demonstrated that, immediately after sorting, CD146-high cells displayed a higher CD44/CD24 ratio and a higher expression of EPcam compared CD146-low cells, in favor of a more pronounced stemness phenotype. In addition, when re-cultured after sorting, CD146-high cells maintained their CD146-high expression while CD146-low cells differentiated into CD146-high and CD146-low cells. Added to the fact that CD146-high cells are the most important population in MDA-MB-231 cells, these results are in favor of the fact that high expression of CD146 constitutes an advantageous phenotype for cells to grow. 

Along this line, the sorted cell populations of CD146-high and CD146-low cells showed that high CD146 expression was correlated with a higher proliferation, migration and invasion capacity. Of interest, soluble CD146 was able to reproduce these effects and it was able to increase many CSC and EMT markers expressed by the CD146-high cells. 

**Figure 8:**
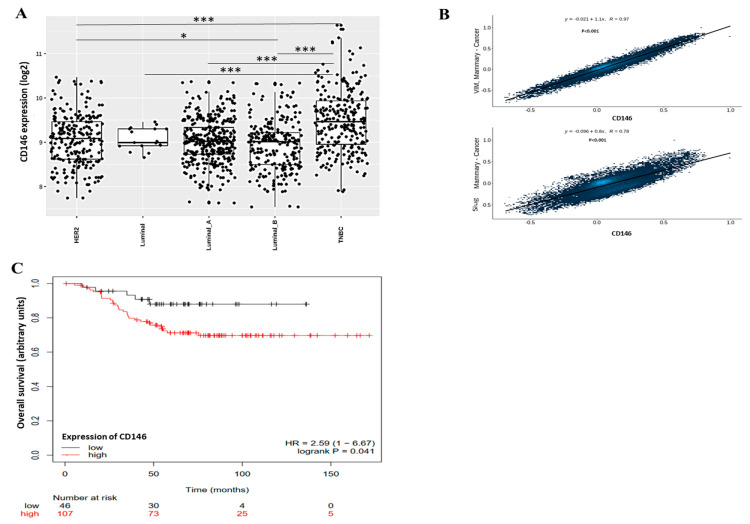
Expression of CD146, correlation with EMT markers and effect on overall survival in patients with breast cancer using public databases. (**A**) CD146 expression was determined in different subtypes of breast cancer, including HER2 cancer, luminal cancer and Triple Negative Breast Cancer (TNBC). Data were analyzed using gent2 in silico tool (http://gent2.appex.kr/gent2; 12 November 2021). (**B**) Expression of EMT markers (Vimentin and Slug) were correlated with CD146 expression in patients with breast cancer. Data were obtained from GitHub repository using correlation AnalyzeR (https://gccri.bishop-lab.uthscsa.edu/shiny/correlation-analyzer; 12 November 2021). (**C**) Overall survival of 153 patients with TNBC was given as a function of the low or high expression of CD146. Data were from the TCGA dataset and analyzed using Kaplan–Meier Plotter tool (https://kmplot.com/analysis/index.php?p=service; 12 November 2021). *, ***: *p* < 0.05, *p* < 0.001, comparison between breast cancer subtypes.

These results will have to be confirmed in other TNBC cell lines expressing high levels of CD146, such as SUM159PT, SUM1315MO2 or SUM149PT cells [31]. In order to compare our results to the literature, we used public databases to estimate the influence of CD146 expression on properties of breast cancer cells and overall survival of patients. Figure 8A shows that CD146 expression is significantly higher in TNBC compared with in other breast cancers, such as HER2 or luminal breast cancers. 

In addition, CD146 expression was significantly correlated with several EMT markers, such as vimentin or slug (Figure 8B). This is in accordance with our results showing that high CD146 MDA-MB-231 cells secrete high levels of sCD146 that, in turn, is able to increase vimentin and slug expression. Finally, clinical studies evidenced a significant decrease in overall survival rate in TNBC patients bearing high CD146 tumors as compared to tumors with weak CD146 expression (Figure 8C). 

This confirms the fact that CD146/sCD146 are relevant markers in this pathology and could represent potential targets for therapy. This is also in accordance with the review of De Kruijff et al. showing that, in univariable analysis, CD146 expression was a prognostic factor for both metastasis-free survival and overall survival [15].

However, it is highly difficult to specifically target CD146 in cancer in general, and in TNBC in particular, essentially because of the high expression of the molecule on surrounding cells, such as the whole vascular system, and of its important physiological functions [2]. In addition, CD146 is able to act through its soluble form generated from the shedding of the membrane form. We, thus, hypothesized that targeting sCD146 could constitute a relevant therapeutic approach. 

This original approach was comforted by the sCD146 effects observed in CD146-high MDA-MB-231 cells. To this end, we thus took advantage of the recent generation of an antibody specifically targeting sCD146, namely the M2J-1 mAb [17]. In our study, this antibody was able to counteract the effects of sCD146 on proliferation, migration and invasion in vitro as well as on CSC and EMT markers both in vitro and in vivo. In addition, it was able to reduce the growth and dissemination of CD146-high MD-MBA-231 cells in two experimental animal models. 

These reported effects of M2J-1 mAb are of major importance since, up to today, the therapeutic options to fight TNBC are very few. The recent development of sacituzumab govitecan that targets Trop2 has increased the survival of patients; however, many mechanisms of resistance to the molecule can appear, mainly through genetic mutations [32]. Immunotherapy has also been shown to be of interest, in particular by combining atezolizumab, an anti-PDL1 antibody, with chemotherapy [33]. We can thus speculate that, in TNBC expressing CD146, combining these therapies with our newly anti-sCD146 antibody M2J-1 could be of therapeutic benefit in order to prevent TNBC dissemination and to increase the overall survival of women attained with this pathology.

## 4. Material and Methods

### 4.1. Cell Culture

Human breast adenocarcinoma-derived MDA-MB-231 cell line was used for the study. The cell line was cultured in RPMI 1640 complete medium (Thermo-Fisher Scientific, Heysham, UK) (RPMI 450 mL + 5 mL PS + 5 mL glutamine + 50 mL FCS), 5% CO_2_ in T-25 flask. The cells were passaged every 3–4 days at 80–90% confluency.

### 4.2. Flow Cytometric Analysis and Cell Sorting

The cultured cells were characterized using Beckman Coulter Gallios Flow Cytometer for cancer stem cell markers CD133 (APC), EPcam (PE), CD24 (Vioblue) and CD44 (FITC). They were also stained for the surface marker CD146 (APC). The two population of CD146 with low and high expression were sorted out using Beckman Coulter MofloAstrios Cell Sorter. The cells were sorted by staining the cells with anti-human CD146 antibody P1H12 for 1 h and then washed with PBS (PBS, no calcium, no magnesium Gibco™). The sorted cell populations were then incubated in CO_2_ incubator for half an hour and then washed with PBS™/™ and used for further analysis.

CD146-high/low cells were sorted with an Astrios sorter (Beckman Coulter, Villepinte, France) according to the manufacturer instructions, and the threshold was established at 2 × 10^4^ for the MFI (Mean Fluorescence Intensity).

### 4.3. Western Blotting

Pierce BCA Protein assay kit (ThermoFisher Scientific) was used for protein analysis. Equal amounts of total protein were resolved on sodium dodecyl sulfate–polyacrylamide gel electrophoresis, transferred onto nitrocellulose membrane by using iBlot™ 2 Gel Transfer Device. The membrane was stained with primary CD146 antibody, incubated overnight and then incubated with horseradish peroxidase conjugated secondary antibody (Santa Cruz Biotechnology, Dallas, TX, USA) before the detection of signals were detected on Syngene GBox.

### 4.4. Cell Proliferation Assay

A total of 5000 FACS-sorted CD146-high/low cells were plated on 96-well plates and incubated for 24 h to achieve 60–70% confluency. After 24 h plating, cells were treated with WST-1 and incubated in CO_2_ incubator for 1 hr. The color intensity was measured using spectrophotometer at 450 nm. In some experiments, the CD146-high cells were treated with the monoclonal antibody targeting sCD146, M2J-1 mAb or with control IgG_1_.

### 4.5. Migration Assay

The FACS sorted CD146-high/low populations of cells were plated on 24-well plates and observed for migration capacity of each population. Around 10,000 cells from each population were incubated overnight to adhere at the surface. The scratch was made using 10 µL tip in each well and kept for 6 h incubation in CO_2_ incubator. Images were taken using microscope (Olympus BX50) at 0 and 6 h after making the scratch. In some experiments, the CD146-high cells were treated with the monoclonal antibody targeting sCD146, M2J-1 mAb or with control IgG_1_.

### 4.6. Cell Invasion Assay

Invasion assays were conducted in BioCoat Matrigel Invasion Chambers (24-well, 8-μm pore, BD Biosciences). In these experiments, the invasion chambers were coated with thin layer of Matrigel and incubated for 1 h in CO_2_ incubator. Meanwhile, the sorted CD146-high and CD146-low cells were counted (approx. 15,000 cells each) and then plated on Matrigel coated invasion chambers. The wells of the 24-well plate were filled with RPMI-1640 media supplemented with 10% serum, acting as a chemoattractant for the invasion of cells. 

The cells were incubated for 24 h, fixed with 4% paraformaldehyde and stained with crystal violet dye. The images were taken under Olympus BX50 microscope. The ability of cells to invade was estimated using image J software. In some experiments, the CD146-high cells were further studied by treating cells with sCD146 concomitantly with the monoclonal antibody targeting sCD146, M2J-1 mAb or with control IgG_1_.

### 4.7. qRT-PCR Experiments

Around 50,000 cells from the total sorted cell population were stored in Trizol. Total RNA was isolated using ReliaPrep™ RNA Miniprep Systems (Promega, Madison, WI, USA) and complementary cDNA was synthesized and analyzed by qRT-PCR using Taqman probes (Thermofisher) for Sox2, Nanog, Slug, Vimentin on Eppendorf^®^ Mastercycler gradient qRT-PCR.

### 4.8. Animal Studies

Nude mice aged between 7–8 weeks were used for the experiment and were kept under strict pathogen free conditions in animal facility. All mice were kept individually and monitored on the daily basis. Two series of experiments were performed. In a first series of experiments, animals were injected subcutaneously with 1,000,000 CD146-high cells and, after the tumors reached about 50 mm^3^, were treated three times a week around the tumor with 500 μg/kg of the monoclonal antibody targeting sCD146, M2J-1 mAb, or with control IgG_1_. The characteristics, conditions of use and safety of the M2J-1 mAb, that specifically targets the soluble form of CD146 without affecting the membrane form, were already described in a previous study [17]. Tumor volume was estimated with calipers for 4 weeks after the beginning of the treatment.

In a second series of experiments, intracardiac injection of 50,000 CD146-high cells was performed along with 500 μg/kg of the monoclonal antibody targeting sCD146, M2J-1 mAb, or with control IgG_1_. After two weeks, mice were sacrificed by cervical dislocation and lungs were extracted. The extracted lungs were kept in 4% paraformaldehyde for 24 h and then transferred to 75% ethanol for 24 h. Blocks of lungs were made using paraffin wax and thin sections were cut using manual microtome.

### 4.9. Histological Staining

The thin sections of the preserved lungs were stained with hematoxylin and eosin to observe the metastasis in the two categories of mice injected with cells, i.e., one/mice injected with CD146-high cells treated with IgG_1_ isotype control and two/mice injected with CD146 high-cells treated with M2J-1 mAb. HE images were taken under the THUNDER Imaging Systems (Leica Microsystems) at different magnifications and clustered and disfigured patches corresponding to metastases were counted in images.

### 4.10. Antibodies and ELISA Kit

The recombinant human soluble form of CD146 corresponding to the shed sCD146 (rh-sCD146) was obtained from Biocytex (Marseille, France). Antibodies used were CD24 (Myltenii Biotech), CD44 (Myltenii Biotech), CD133 (e-Biosciences), EPcam (Biolegend), CD146 (Biocytex), Sox 2 (Santa Cruz), Nanog (R&D), Slug (Cell Signaling), Vimentin (Cell Signaling) and Actin (Biolegend) ELISA kit for measuring sCD146 was from Biocytex (Marseille, France). The anti-sCD146 monoclonal antibody M2J-1 was used [17].

### 4.11. Public Databases

We utilized the gent2 (http://gent2.appex.kr/gent2; 12 November 2021), GitHub repository (https://gccri.bishoplab.uthscsa.edu/shiny/correlation-analyzer; 12 November 2021) and TCGA dataset [Breast] (https://kmplot.com/analysis/index.php?p=service; 12 November 2021) public databases to further investigate the correlation between CD146 expression and the type of breast cancer, the correlation between CD146 and Epithelial to Mesenchymal (EMT) markers and the overall survival of patients with Triple Negative Breast cancer (TNBC), respectively.

### 4.12. Statistical Analysis

The data are expressed as the mean ± SEM of the indicated number of experiments. Statistical analysis was performed with the Prism software (GraphPad Software Inc., San Diego, CA, USA). Significant differences were determined using a non-parametric Mann Whitney test in both in vitro and in vivo experiments when comparing two groups. A *p*-value < 0.05 was considered as significant.
